# Influence of LRP5 (rs556442) polymorphism on insulin resistance in healthy Iranian children and adolescents

**DOI:** 10.3906/sag-1809-107

**Published:** 2019-04-18

**Authors:** Nima MONTAZERI-NAJAFABADY, Mohammad Hossein DABBAGHMANESH, Rajeeh MOHAMMADIAN AMIRI, Marzieh BAKHSHAYESHKARAM, Gholamhossein RANJBAR OMRANI

**Affiliations:** 1 Endocrinology and Metabolism Research Center, Nemazee Hospital, Shiraz University of Medical Sciences, Shiraz Iran; 2 Health Policy Research Center, Shiraz University of Medical Sciences, Shiraz Iran

**Keywords:** LRP5, insulin, restriction fragment length polymorphism, revised McAuley index, children

## Abstract

**Background/aim:**

Genetic aspects play a role in insulin resistance in children. In this study, for the first time, the association of LRP5 (rs556442) polymorphism and insulin resistance in Iranian children and adolescents was investigated.

**Materials and methods:**

The study population comprises children and adolescents aged 9–18 years. Anthropometric and biochemical parameters were assessed. Insulin resistance/sensitivity was determined by the quantitative insulin sensitivity check index (QUICKI), homeostasis model assessment-insulin resistance (HOMA-IR), insulin-to-glucose ratio, McAuley index, revised McAuley index, fasting insulin resistance index (FIRI), and Bennett’s index. LRP5 (rs566442) single nucleotide polymorphism (SNP) was identified using restriction fragment length polymorphism (RFLP). Linear regression analysis was used to determine the association between the LRP5 polymorphism (rs556442) and insulin sensitivity indexes.

**Results:**

Significant differences were found between GG genotype vs. AG/AA genotypes for McAuley index (P = 0.049) and revised McAuley index (P = 0.044) when adjusted for interaction factors (age, sex, and puberty) in regression models. No significant association was found between LRP5 (rs566442) and other insulin resistance indexes. Also, LRP5 (rs566442) did not show a significant impact on biochemical parameters.

**Conclusion:**

This study showed that LRP5 polymorphism (rs556442) was associated with insulin resistance in Iranian children and adolescents.

## 1. Introduction 

Insulin resistance (IR) is mostly related to metabolic disorders such as obesity, metabolic syndrome (MS), hypertension, type 2 diabetes, chronic hepatitis C, and ischemic cardiovascular disease (1–7). IR is defined as defective insulin action in decreasing blood glucose, according to failing physiological response to insulin (6,8). In the pediatric and adolescent age group, insulin resistance is known as a serious health problem associated with obesity, dyslipidemia, cardiometabolic risk, and inflammation (1,2). Identifying children with insulin resistance is a good strategy for preventing high-risk children from MS interventions (9).

Various methods have been validated for direct and indirect assessment of insulin resistance in clinical and epidemiological studies (10). Hyperinsulinemic–euglycemic clamp as a direct method is a gold standard for this purpose, but it is expensive and invasive (1,6). Indirect methods derived from fasting insulin and glucose include intravenous glucose tolerance test, oral glucose tolerance test, meal tolerance test, homeostatic model assessment of insulin resistance (HOMA-IR), quantitative insulin sensitivity check index (QUICKI), insulin-to-glucose ratio, fasting insulin resistance index (FIRI), and Bennett’s index (1,2). Two other indexes based on insulin and triglyceride (TG), the McAuley index (11) and body mass index (BMI), and index based on triglyceride, the revised McAuley index, were also used to determine insulin resistance in research/epidemiological studies (10). HOMA-IR is a marker of insulin resistance and beta cell function that is a noninvasive method used to compare insulin secretion and insulin resistance (12,13). The McAuley index, as reported in various studies, is a precise process with better reproducibility to measure IR than other indexes (14).

Genetic aspects play a role in insulin resistance in both adults and children by creating alterations in the pathways related to insulin metabolism (15). The Wnt signaling pathway is one of the systems that interacts and modulates the insulin-signaling network (16). Low-density lipoprotein (LDL) and receptor-related protein (LRP5) as a cell membrane receptor have a critical role in the Wnt signaling pathway (17). Recent studies suggested that there was a crosstalk between the insulin and Wnt signaling pathways. This relationship occurs at different levels and in different cellular contexts (16). In addition, both insulin and insulin-like growth factor-1 (IGF-1) stimulation activate the Wnt signaling pathway in the hepatoma cells (16). Another study showed that LRP5 was required for normal insulin signaling in preadipocytes (16). This pathway is involved in various cellular activities like embryonic development, oncogenesis, myogenesis, adipogenesis, and glucose homeostasis (17,18). The Wnt pathway regulates prenatal β-cell development and postnatal β-cell functions, and its receptor, LRP5, is highly expressed on adult pancreatic β-cells (19). It is essential for glucose-induced insulin secretion (17). LRP5-deficient mice showed impaired glucose tolerance as a result of impaired insulin production from β-cells in the pancreas (20). Polymorphisms and mutations in Wnt and its components such as LRP5 and LRP6 may lead to increased risks of obesity, osteoporosis, and metabolic syndrome (16). However, the relationship of LRP5 (rs556442) polymorphism with insulin sensitivity and insulin resistance in children has not been examined as yet. For this reason, in this paper we investigated whether LRP5 (rs556442) polymorphism is associated with insulin resistance in Iranian children and adolescents.

## 2. Materials and methods

### 2.1. Study population

In this cross-sectional study, which was performed in Kawar, an urban area located 50 km east of Shiraz, the capital city of Fars Province in the south of Iran, healthy Iranian children and adolescents aged 9–18 years were enrolled. Five hundred children and adolescents (250 girls and 250 boys) were selected using age-stratified systematic random sampling. Among them, 266 children and adolescents were randomly selected for the genetic study. The children known to have systemic disease (e.g., thyroid problems, diabetes, renal failure, adrenal insufficiency), history of precocious or delayed puberty, or using medications (e.g., anticonvulsants or steroids) were not included in the study. Our study was approved by the ethics committee of Shiraz University of Medical Sciences. An informed consent form was obtained from each participant or their parents.

### 2.2. Anthropometry

Height and weight of the children were measured using a wall-mounted meter and standard scale (Seca, Germany), respectively. Height was rounded to the nearest 0.5 cm and weight to the nearest 0.1 kg. BMI was calculated by this formula: BMI (kg/m2) = weight (kg) / [height (m)] 2. Children’s pubertal stages were assessed by an endocrinologist and bone density was measured by dual-energy X-ray absorptiometry (DEXA). Pubertal stage was evaluated according to the Tanner standard classification during the visit for the DEXA scan. Children with Tanner stages of 1 were considered as prepubertal, 2 and 3 as early pubertal, and 4 and 5 as pubertal.

### 2.3. Laboratory tests

All the blood samples were taken in the Shiraz Endocrinology Research Center after overnight fasting. Serum total cholesterol, high-density lipoprotein (HDL-C), fasting blood sugar (FBS), and TG concentrations were measured by enzymatic reagents (Biosystems, Barcelona, Spain) with an A-25 Biosystems autoanalyzer. The serum insulin concentration was measured with a radioimmunoassay kit (IZOTOP, Budapest, Hungry). In addition, insulin resistance/sensitivity was determined by the QUICKI, HOMA-IR, insulin-to-glucose ratio, McAuley, revised McAuley, FIRI, and Bennett’s indexes and calculated using the following formulas (21):

QUICKI: 1 / log (glucose mg/dL) + log (insulin µU/mL)

HOMA-IR: (fasting insulin [microunits per milliliter] × fasting glucose [millimoles per liter]) / 22.5

Insulin-to-glucose ratio: Insulin (µU/mL) / glucose (mmol/L) 

McAuley: exp [2.63 – 0.28ln (insulin) − 0.31ln (triglyceride)]

Revised McAuley: exp [3.29 – 0.25ln (insulin) – 0.22ln (BMI) – 0.28ln (triglyceride)]

FIRI: Insulin (µU/mL) × glucose (mmol/L) /25

Bennett’s index: 1 / log [glucose (mmol/L)] × log [insulin (µU/mL)]

### 2.4. Genotyping

LRP5 (rs566442) single nucleotide polymorphism (SNP) was identified using PCR/RFLP. The details of primer sequences and PCR conditions were mentioned in our previous study (22). This SNP (A→G) was located 123 bp downstream of the starting site in exon 15 of the LRP5 gene. The genomic DNA was extracted from the peripheral blood with the DNA QIAamp blood kit (QIAGEN, Hilden, Germany), according to the manufacturer’s instructions.

### 2.5. Statistical analysis 

Data for all variables are shown as means and standard deviations (SDs) or medians and interquartile ranges. For data with a normal distribution, t-test and ANOVA, and for data with nonnormal distribution Mann–Whitney and Kruskal–Wallis tests were used to evaluate the differences between the genotype groups. The chi-square (χ2) test or Fisher’s exact test was used to determine the genotype distribution and allele frequencies. Nonnormal data were log-transformed before analysis. Linear regression analysis was performed in 2 models to find the association between insulin resistance/sensitivity indexes and genotype groups (dominant and recessive) when adjusted for interacting factors (age, sex, and puberty). For the dominant genetic model, the GG genotype was set as the reference and compared to AA+AG genotypes. For the recessive genetic model, the AG+GG genotype was set as the reference and compared to the AA genotype. Model 1 was adjusted for age and sex, and model 2 for age, sex, and puberty. P**values less than 0.05 were considered statistically significant. All statistical analyses were performed using IBM SPSS Statistics 22.0 for Windows (IBM Corp., Armonk, NY, USA).

## 3. Results 

### 3.1. Clinical and biochemical characteristics

A total of 266 children (139 girls, 127 boys) aged 9–18 years (mean: 13.64 ± 2.86) were enrolled in the study. The means and SDs for age, weight, height, BMI, fasting blood sugar, serum insulin concentration, triglycerides, total cholesterol, LDL-C, HDL-C, HOMA-IR, QUICKI, Bennett’s, McAuley and revised McAuley, FIRI, and insulin-to-glucose ratio are shown in Table 1.

**Table 1:** General characteristics of the study population.

Variable	Mean ± SD	Median (interquartile range)
Age	13.6 ± 2.86	14 (4)
Height	152.5 ± 14.75	154 (20)
Weight	41.65 ± 13.64	42 (20)
BMI	17.51 ± 3.34	17.2 (4.3)
Cholesterol	156.05 ± 30.44	153 (39)
HDL	47.3 ± 15.28	45.5 (15.9)
TG	70.6 ± 45.65	62 (57)
LDL	94.6 ± 27.12	93.3 (34.8)
Fasting blood sugar	78.3 ± 12.41	77 (16)
Insulin	8.9 ± 5.33	7.7 (3.6)
QUICKI	1.4 ± 0.24	1.4 (0.20)
HOMA-IR	1.7 ± 1.24	1.4 (0.83)
Insulin-to-glucose ratio	2.1 ± 1.12	1.7 (0.98)
McAuley	2.3 ± 0.73	2.1 (0.87)
Revised McAuley	2.6 ± 0.61	2.5 (0.70)
FIRI	1.5 ± 1.12	1.3 (0.75)
Bennett’s index	1.4 ± 0.39	1.3 (0.40)

### 3.2. Genotyping 

The studied SNPs were in Hardy–Weinberg equilibrium (P = 0.71) and showed a minor allele frequency (MAF) higher than 10%, representing a good penetration of the alleles in the population. LRP5 (rs566442) genotype distribution was 41.2% for GG, 45.4% for AG, and 13.4% for AA in the whole population. There were no differences between the girls and boys (P = 0.47). The total allelic frequencies were 0.59 for G and 0.41 for A. In boys, allele frequency was 34.7% and 65.3% for A and G, respectively. In girls, it was 37.6% for A and 62.4% for G. There were no significant differences between allele distribution and sexes (P = 0.35). The genotype distribution regarding sex is illustrated in Table 2.

**Table 2:** Genotype counts and frequencies of the rs566442 polymorphism.

	Boys	Girls	P-value
Genotypes			
GG	58 (42.3%)	50 (40%)	
AG	63 (46%)	56 (45%)	0.47
AA	16 (11.7%)	19 (15.2%)	
Alleles			
G	179 (65.3%)	156 (62.4%)	0.35
A	95 (34.7%)	94 (37.6%)	

### 3.3. Effects of rs566442 polymorphism on insulin indexes

The effect of rs566442 polymorphism on insulin function is shown in Table 2. No significant association was found among LRP5 genotype and fasting blood glucose, serum insulin concentration, QUICKI, Bennett’s index, HOMA-IR, FIRI, insulin-to-glucose ratio, and McAuley and revised McAuley indexes. However, children with the AA genotype showed lower values of serum insulin concentration, QUICKI, Bennett’s, HOMA-IR, FIRI, and insulin-to-glucose ratio. 

### 3.4. Association of rs566442 polymorphism with McAuley and revised McAuley indexes

Linear regression analysis showed a significant association between revised McAuley index in the dominant (model 1: P < 0.0001, model 2: P < 0.001) and recessive genetic models (model 1: P = 0.03, model 2: P = 0.03). No significant association was observed in McAuley index or rs566442 polymorphism in the dominant and recessive genetic models (Tables 3, 4).

**Table 3:** Effect of the LRP5 (rs566442) polymorphism on insulin function indices.

Data	GG (n = 109)	GenotypeAG (n = 119)	AA (n = 36)	P-value
		Mean (SD)		
Fasting blood sugar	77.83 (12.36)	78.55 (13.11)	79.33 (10.19)	0.73
Insulin	8.96 (5.05)	9.20 (6.01)	8.14 (3.61)	0.79
QUICKI	1.42 (0.23)	1.42 (0.23)	1.37 (0.28)	0.75
HOMA-IR	1.76 (1.19)	1.84 (1.41)	1.60 (0.75)	0.79
Insulin-to-glucose ratio	2.08 (1.06)	2.09 (1.24)	1.86 (0.88)	0.60
McAuley	2.48 (0.86)	2.24 (0.60)	2.20 (0.70)	0.08
Revised McAuley	2.82 (0.76)	2.58 (0.47)	2.45 (0.44)	0.07
FIRI	1.58 (1.07)	1.65 (1.27)	1.44 (0.67)	0.79
Bennett’s index	1.43 (0.38)	1.41 (0.38)	1.33 (0.46)	0.53

**Table 4:** Linear regression analysis for association of McAuley and revised McAuley indexes in genotype groups in 2 models. P values less than 0.05 are shown in bold.

Data	McAuley index					
	Model 1			Model 2		
	OR (CI 95%)		P-value	OR (CI 95%)		P-value
LRP5 (rs556442)						
Dominant (AA + AG) vs. GG	–0.25 (–0.55 to 0.49)		0.9	–0.22 (–0.53 to 0.1)		0.17
Recessive AA vs. (AG + GG)	–0.25 (–0.55 to 0.56)		0.1	0.02 (–0.45 to 0.5)		0.9
	Revised McAuley index					
		Model 1			Model 2	
	OR (CI 95%)		P-value	OR (CI 95%)		P-value
LRP5 (rs556442)						
Dominant (AA + AG) vs GG	–0.27 (-0.42 to 0.12)		<0.0001	–0.26 (–0.4 to –0.1)		<0.001
Recessive AA vs (AG + GG)	–0.23 (–0.4 to 0.1)		0.03	–0.24 (–0.4 to 0.1)		0.03

Model 1: Adjusted for age and sex. Model 2: Adjusted for age, sex and puberty.

## 4. Discussion 

Insulin resistance is linked to the pathophysiologic pathways that originate during childhood. Conversely, an individual’s genetic background variation may possibly cause alterations in these pathways, which leads to disease progression (23). In addition to genetic factors, metabolic factors such as obesity and abdominal fat deposition impact insulin resistance (13). Several studies on twins showed 50% change in insulin sensitivity and secretion in relation to genetic factors (1). Another study suggested that healthy children with a family history of type 2 diabetes in comparison to those without it were possibly insulin-resistant (1).

In children and adolescents, IR is not numerically defined and one biochemical parameter that can explain IR fully and accurately does not exist. The IR threshold for prediction of IR is not known. No acceptable cut-off value with high accuracy is available. Normative data for distribution of different biochemical markers in childhood do not exist (24). 

In this study, we evaluated the association of LRP5 (rs566442) polymorphism with insulin resistance indexes HOMA-IR, QUICKI, insulin-to-glucose ratio, McAuley, revised McAuley, FIRI, and Bennett’s index. To the best of our knowledge, this is the first study to investigate the association of LRP5 and insulin resistance in healthy children with different defined criteria.

In this study, we found a significant association of LRP5 (rs566442) polymorphism with revised McAuley index. According to the regression analysis, there was a significant difference in the revised McAuley index between GG and AG/AA genotypes (dominant model), and AA and AG/GG genotypes (recessive model). However, we did not find this association between this genetic variation and other insulin resistance indexes. As reported previously, the McAuley and revised McAuley are IR measurement indexes, which better estimate IR among normoglycemic individuals (25). In that study, correlation coefficients of the 5 indices showed that the McAuley index had the strongest correlation with IR (25). Ascaso et al. observed that the McAuley index was more specific and sensitive in the assessment of IR compared to QUICKI (26). Another study on the nondiabetic offsprings of diabetic individuals found the highest AUC for metabolic syndrome detection by using the McAuley index (27).

Carg et al. observed that the McAuley index had the highest specificity in detecting metabolic syndrome in urban Indian adolescents (14). In the McAuley and revised McAuley index, insulin resistance is determined by measures based on fasting insulin and triglyceride levels.

In this study, we observed that individuals with the GG genotype had significantly higher revised McAuley index scores compared to AA/AG genotypes. The McAuley and revised McAuley are IR measurement indexes that better reflect the lipid profile (28). They were inversely correlated with TG levels. Many previous studies have confirmed that IR and the accompanying hyperinsulinemia can directly affect the lipoprotein metabolism (29). Increased TG levels, as a result of hyperinsulinemia, also directly cause pancreatic beta-cell dysfunction due to the accumulation of TG inside the cells (25). Based on these data, IR indexes such as the McAuley and revised McAuley could be a good reflection of dyslipidemia, which occurs due to metabolic dysfunction and hyperinsulinemia (25).

A few studies have investigated the role of the Wnt signaling pathway and LRP5 in glucose and insulin secretion. Fujino et al. observed that glucose-induced insulin secretion was conducted by LRP5 in mice (17). They also concluded that LRP5 controls the normal function of the β-cells through the transcriptional regulation of Tcf1, Tcf2, and HNF-4α genes (17). Palsgaard et al. found that the Wnt signaling pathway in 3T3-L1 preadipocytes causes phosphorylation of Akt, ERK1/2, and GSK3 proteins involved in insulin signal transduction (16). Decrease in LRP5 expression also has a negative effect on adipocyte differentiation and triglyceride accumulation because of the inhibition of the adipogenesis process by Wnt signaling (16). Several interesting traits were found about the role of LRP5 in insulin signaling. First, LRP5 markedly affects the basal phosphorylation of insulin signaling proteins and the insulin response in preadipocytes. Second, this is a mechanism that is especially related to insulin as compared with IGF-1 in 3T3-L1 cells. Third, LRP5 has positive effects on insulin signaling via interaction between LRP5 and insulin receptor (16). These findings show that LRP5 plays an important role in insulin signaling through interaction with insulin receptor induced by phosphorylation of insulin signaling proteins (16). It was observed that mutations in LRP5 were significantly associated with diabetes or impaired glucose tolerance in a family-based study and this phenotype occurred as the result of reduction in LRP5 expression, which may lead to insulin resistance in preadipocytes, varying the balance of the pro- and antiadipogenic signals and changing glucose metabolism (30). In another study, individuals with homozygous or heterozygous LRP5 mutations (R570W) revealed impaired glucose metabolism with high frequency, but there was no evidence of insulin resistance in the subjects (31). However, LRP5 (R1036Q) mutation displayed obvious impaired beta-cell function by intravenous glucose tolerance test (31). Kim et al. found that dysregulation of TG may be the main factor of early stage IR. Elevation of glucose concentration in the hepatocytes beyond its capacity to convert glucose to glycogen results in an increase in the insulin secretion. Insulin converts the excess glucose to free fatty acids, and finally these molecules are accumulated as TG. Thus, TG accumulation represents hyperinsulinemia (25). In the current study, we found that individuals carrying the mutant allele compared to those who did not carry this allele had significantly higher insulin resistance. 

Our study had some limitations. Due to the relatively small sample size, statistical power to detect associations of LRP5 variants with insulin resistance was limited. Subjects enrolled in the present study were selected from a cohort study from the south of Iran and may not represent the general population of Iranian children. More studies need to be carried out in Iranian and other population groups to understand the correlation between LRP5 polymorphism and insulin resistance to further verify our results. Furthermore, the larger sample size would help to elucidate the association of LRP5 with insulin resistance and other metabolic diseases.

In conclusion, this study showed that LRP5 polymorphism (rs556442) was associated with insulin resistance in Iranian children. However, further studies should be conducted to evaluate the role of the other polymorphisms.

## Acknowledgments

The authors would like to thank Dr Nasrin Shokrpour for editorial assistance. This study was funded by project No.33-2467 and approved by the Shiraz University of Medical Sciences.
